# The British Medical Association at Bristol

**Published:** 1894-08-18

**Authors:** 


### THE BRITISH MEDICAL ASSOCIATION AT BRISTOL.

### THE ANNUAL MUSEUM.

(FIRST NOTICE.)

This exhibition was decidedly one of the most successful features of the Bristol meeting. A very large handsome drill hall, close to the rooms of the sections, was entirely filled with rows of stalls. On these over a hundred exhibitors displayed a collection of foods, drugs, instruments, and books such as had never been shown at any previous meeting. Both local and other firms were well represented, and a most enjoyable conversazione was given by the exhibitors on the Tuesday evening in the hall itself, when the splendid band of the Royal Marine Artillery played till a late hour. The excellence of the exhibits and the enterprise of the exhibitors was very striking, but we cannot help wishing that arrangements had been made to admit the general public for one afternoon. Regrets, too, were felt that the stalls were dismantled so early on Friday when many visitors were free for the first time to study this invaluable collection of surgical appliances.

Messrs. FERRIS AND Co. occupied a large space with a brilliant exhibition which did credit to their well-earned and world-wide reputation. The brief enumeration of their exhibits occupies six pages in the official catalogue. Prominent among them was Mr. Greig Smith's operating-table, a model of simplicity and strength, which attracted much attention on all si es. It shows remarkably few crevices in which dust may harbour. It appears to be built entirely of nickelled steel and glass, and is readily altered to suit the various operations of modern surgery. From its convenience and the easiness with which it can be purified it will be widely used. The "Ever-ready Plaster Caddy," which has been devised for keeping spread plasters and lints in a clean and handy form ready for use, is another contrivance well worthy of attention. There was a fine selection of drugs, and we noticed no less than six preparations of thyroid products varied to meet the taste of the most fastidious. Messrs. Ferris were among the first to prepare thyroid juice, and to the excellence of their work much of the success is due which has followed its use. Other organic extracts are now shown by them. The busy practitioner will find their "Perpetual Visiting List and Day-book" combined in a thin compact little case to be a great saving of time. A tiny match-box is made to hold sublimate powders, each of which suffices to produce a pint of $\frac{1}{1000}$ solution. These powders are very handy for the surgeon's bag, and save the risk of carrying bottles. We must not forget to notice a portable case devised by Dr. Michell Clarke with reagents and apparatus for detecting micro-organisms in the secretions. This is a great help not only to those whose work has to be done in different places, but also in every case where immediate tests have to be applied at the bed side. Another case is that for medical officers of health devised by Dr. G. Dyer. Very convenient and portable.

Defries and Son showed specimens and models of the Pasteur Filter, for which they are agents. These filters appear to be essentially earthenware cones such as are used in bacteriological work, and are sold in a variety of useful shapes.

The Chemists' Ærated and Mineral Waters Association had a beautiful stall, where the "Camwall" Gold Medal Table Waters were exhibited. They offer a soda water of guaranteed strength for prescribing purposes, as well as many varieties of table waters of reliable character.

Mr. Henri Nestle exhibited at an artistic stall the Nestle's food for invalids and infants and Nestle's Swiss Milk. These twoa rticles are so well known to the medical profession that comment is unnecessary.

Messrs. Salmon, Ody, and Co. made an excellent display of trusses, the plates being made of aluminium, saving two-thirds of the weight to the wearer. They also had another new feature in their patent trusses, viz., the springs covered with xylonite. The remainder of the truss is aluminium, with ordinary leather pads, making thoroughly rust-proof springs, noticeable for extreme lightness. Their exhibit of artificial limbs, belts, and various surgical appliances was of excellent finish and great interest.

Another old acquaintance was the Hamburg Hoff's Malt Extract stall, with various delicious preparations.

The Sanitary Wood Wool Company showed their dressings, in the make of which some great improvements have been made. Their efficacy and cheapness has gained for them a very wide sale; more than 200,000 are used annually. We were much interested in a valuable invention of theirs—a patent drainage tube, consisting of catgut woven together so as to form a hollow openwork tube of any suitable size. These tubes are permeable throughout, aseptic, light, and not irritating. Their accouchement outfits are very largely used, and contain a complete set of aseptic articles necessary for confinements.

Harker, Stagg, and Morgan, among other palatable preparations, exhibit their Horseshoe Brand Cascara Jelly, in which the bitter taste of the drug is removed without, it is said, in any way affecting its medicinal qualities. It forms a very elegant preparation and will doubtless be a great favorite.

Arthur and Co., 69, Berners Street, W., have many new preparations made in accordance with the suggestions of eminent members of the medical profession. We can only notice their Liq. Tannici Co., an anhydrous tincture for throat sprays, dermatitis, &c.; the antiseptic handkerchiefs made of soft tough paper. They are to be burnt after use. In phthisis, measles, and even in ordinary colds we strongly recommend them as an important prevention against the spread of disease. The ordinary handkerchief should never be allowed to consumptive patients. They show specimens also of microscopic staining fluids with which especial care has been taken to prevent precipitation, and to render them permanent.

Alex. Riddle and Co. had an attraction in their celebrated Stowers Lime Juice Cordial and kindred preparations famed for their freedom from mustiness. This cordial, whether as a substitute for, or as an addition to alcoholic beverages, has a great demand, and this was shown by the interest taken in it by those attending the exhibition.

Corbyn, Stacey, and Co., had a variety of their pharmaceutical preparations, elegant in appearance and taste.

Clark's Bread Company showed their "Ne Far" biscuits recently recommended by Dr. Saundby for diabetics. They are pleasant to eat and a great improvement on ordinary gluten bread.

Mr. L. Rawsthorn's Limona Food, a digestive and regulative diet for the use of invalids and children, was attractively placed near the main entrance. This food, which contains all the naturally valuable matter of the wheat grain is rendered still more valuable by the combination of hypophosphites of iron, lime, and soda in approved proportion. It is easily prepared and can be made into excellent puddings.

Messrs. Dowie and Marshall's boots and shoes are well known. Nothing is more strange than the difficulty there is to get ordinary bootmakers to fashion a boot with the inner edge of the sole forming a straight line. Messrs. Dowie and Marshall not only offer shoes without this defect but show an instrument for drawing back distorted great toes to their proper original direction.

The Longford Wire, and Steel Company excited interest by the exhibition of one of their bedsteads and mattresses loaded with a ton of iron weights, which it bore most unconcernedly with apparent ease.

Bishop and Son, sanitary engineers, Bristol, were noticeable for their "Gem" Automatic apparatus for disinfecting closets and lavatories. This invention adds a certain quantity of fluid disinfectant to each flush of water and is of great use, especially where infective cases are nursed.

Maggs and Co., Clifton, draw attention to their purified bedding. Quantities of filthy and infected rags are made up by unscrupulous traders into flock for mattresses, and it is of the highest importance to find firms who will guarantee that all their materials are purified by the only effective methods, steam and dry heat.

Next to their stall was that of Gale and Sons and Whitfield and Co., where the Lawson Tait spring bedsteads were to be seen.

Alfred Carter (Limited) had a most interesting display of invalid chairs and reading machines. We have been quite astonished at the ease with which infirm patients propel themselves in these wheel chairs, and the adjustable couches for spinal cases are a marvel of ingenuity and comfort to the patients.

The Aylesbury Dairy Co. showed their "Humanized Milk," perfectly sterilized, of great use where mothers' milk cannot be obtained, also ordinary sterilized milk for travellers, and "Mufflers" food for infants after weaning, and other preparations.

The epileptic bedsteads shown by Billington Bros., Liverpool, are of important to managers of asylums and those patients who suffer from nocturnal convulsions.

At Messrs. Kepple's stall, Bristol, we noticed the "Wittmann" patent filter which was praised by Mr. H. M. Stanley. Their glass and artistic chinaware is of great beauty.

The revolution which antiseptic surgery has effected in the instruments and fittings of hospital theatres is well brought out by the exhibits of Messrs. Down Bros. The latest patterns entirely of glass and metal were on view and made a brilliant show. The finish and perfection of the apparatus are striking.

Messrs. Allen and Hanburys had one of the largest exhibits in the hall, and one which repaid careful study. We shall hope to say more of some of their specimens hereafter. Their Malt Extract, Bynin, Perfected Castor Oil, Tabellæ, Soluble Gelatine Coated Pills, and Malted Infant's Food are too well known to need more than a passing reference. Their surgical instruments and vaporizers were shown in great variety and attracted much attention. Here, too, were a number of preparations shown by Mr. T. Buxton, Clifton. His Pepsin and Bismuth mixtures of all kinds were well worth notice, and his chloride of ammonium inhaler was remarkable for the absence of any indiarubber tubing. Pepsin preparations and powders were accumulated at this spot, since Armour and Co. exhibited close to the last stall a selection of their numerous high testing digesting ferments. The bookstalls were an important feature. The English and American publications shown by The Scientific Press included a list of the company's new works on medical, scientific, and institutional subjects, as well as their weekly and monthly periodicals, and the Reports of the Johns Hopkins Hospital, for which the Press are English agents.

*(To be continued.)*

